# Mucin Variable Number Tandem Repeat Polymorphisms and Severity of Cystic Fibrosis Lung Disease: Significant Association with *MUC5AC*

**DOI:** 10.1371/journal.pone.0025452

**Published:** 2011-10-06

**Authors:** XueLiang Guo, Rhonda G. Pace, Jaclyn R. Stonebraker, Clayton W. Commander, Anthony T. Dang, Mitchell L. Drumm, Ann Harris, Fei Zou, Dallas M. Swallow, Fred A. Wright, Wanda K. O'Neal, Michael R. Knowles

**Affiliations:** 1 Cystic Fibrosis/Pulmonary Research and Treatment Center, University of North Carolina at Chapel Hill, Chapel Hill, North Carolina, United States of America; 2 Departments of Pediatrics and Genetics, Case Western Reserve University, Cleveland, Ohio, United States of America; 3 Human Molecular Genetics Program, Children's Memorial Research Center, Northwestern University Feinberg School of Medicine, Chicago, Illinois, United States of America; 4 Department of Biostatistics, University of North Carolina at Chapel Hill, Chapel Hill, North Carolina, United States of America; 5 Research Department of Genetics, Evolution and Environment, University College London, London, United Kingdom; 6 Gene Modifier Study Group, Chapel Hill, North Carolina, United States of America; University of Colorado Denver, United States of America

## Abstract

Variability in cystic fibrosis (CF) lung disease is partially due to non-*CFTR* genetic modifiers. Mucin genes are very polymorphic, and mucins play a key role in the pathogenesis of CF lung disease; therefore, mucin genes are strong candidates as genetic modifiers. DNA from CF patients recruited for extremes of lung phenotype was analyzed by Southern blot or PCR to define variable number tandem repeat (VNTR) length polymorphisms for *MUC1*, *MUC2*, *MUC5AC*, and *MUC7.* VNTR length polymorphisms were tested for association with lung disease severity and for linkage disequilibrium (LD) with flanking single nucleotide polymorphisms (SNPs). No strong associations were found for *MUC1*, *MUC2*, or *MUC7*. A significant association was found between the overall distribution of *MUC5AC* VNTR length and CF lung disease severity (p = 0.025; n = 468 patients); plus, there was robust association of the specific 6.4 kb *Hinf*I VNTR fragment with severity of lung disease (p = 6.2×10^−4^ after Bonferroni correction). There was strong LD between *MUC5AC* VNTR length modes and flanking SNPs. The severity-associated 6.4 kb VNTR allele of *MUC5AC* was confirmed to be genetically distinct from the 6.3 kb allele, as it showed significantly stronger association with nearby SNPs. These data provide detailed respiratory mucin gene VNTR allele distributions in CF patients. Our data also show a novel link between the *MUC5AC* 6.4 kb VNTR allele and severity of CF lung disease. The LD pattern with surrounding SNPs suggests that the 6.4 kb allele contains, or is linked to, important functional genetic variation.

## Introduction

Mucin glycoproteins are critically important in airway epithelial biology and innate immunity [1]. For most mucins, the highly glycosylated tandem repeat (TR) domain shows variation in repeat number, and are referred to as VNTRs (variable number tandem repeats) [2], [3]. Large differences in VNTR sizes may alter mucin protein molecular weight by as much as two-fold [4].

In the airways, secreted mucins (e.g. MUC5AC and MUC5B) are key for mucociliary clearance, and transmembrane mucins (e.g. MUC1 and MUC4) contribute to glycocalyx barrier function [1], [2]. Respiratory diseases, including cystic fibrosis (CF), asthma, and chronic obstructive pulmonary disease (COPD), have recognized alterations in normal mucin expression/properties that contribute to pathophysiology [2]. In CF, defective ion transport leads to dehydration of the airway surface liquid, reduced mucociliary clearance, and susceptibility to chronic lung infections [5].

Lung disease severity varies widely in CF, and at least 50% of this variability is heritable [6]. Several genetic modifiers of CF lung disease severity have been identified, but do not explain all of the heritable variation [7]–[9]. Polymorphisms in respiratory mucins could contribute to modifier effects, particularly variation in VNTR length, which cannot be directly queried on panels used for genome-wide association studies (GWAS).

We tested for association between CF lung disease severity and VNTR length polymorphisms in mucin genes expressed in the airway, which have been shown to have VNTRs. These were *MUC1*, *MUC2*, *MUC5AC* and *MUC7* whose encoded proteins are of recognized importance in normal airway defense, and/or prior studies that indicate they play a role in diseases of the lung, and other inflammatory or malignant disease. There are reported associations with VNTR length for *MUC1* in lung adenocarcinoma, H.pylori gastritis and gastric carcinoma [10]–[12]; and *MUC2* and *MUC7* with asthma [13]–[15]. *MUC5AC* was chosen since it encodes one of the two key secreted airway mucins that has a VNTR [16], [17], and it was hypothesized that this variation may play an important, but as yet unidentified, role in airway disease [1]. The other important airway secreted mucin, encoded by *MUC5B*, has no common VNTR variation and thus was not tested in this study. Using Southern Blot methods that define *MUC5AC* allele lengths more precisely than previously reported [17]–[18], we identified robust association of a specific *MUC5AC* VNTR allele (6.4 kb, *Hinf*I-digested DNA) and severe CF lung disease. For *MUC5AC* and *MUC1*, we demonstrated significant linkage disequilibrium (LD) between neighboring SNPs and VNTR alleles categorized into size modes. Yet, even within modes, patterns of association could separate alleles, and the *MUC5AC* 6.4 kb VNTR allele was distinct from the 6.3 kb VNTR allele. These findings highlight the complexity of genetic variation around mucin VNTR regions and emphasize the need for further studies of individual polymorphic alleles.

## Methods

Additional detail is provided in the supporting information (Text S1).

### Ethics statement

This study was conducted with the approval of the University of North Carolina at Chapel Hill Institutional Review Board and informed written consent was received from all patients. The manuscript does not contain identifying patient information. The data were analyzed anonymously and all clinical investigations have been conducted according to the principles expressed in the Declaration of Helsinki.

### Patient population

CF patients (Phe508del homozygotes), enrolled from extremes of lung phenotype (“severe” and “mild”), were studied (Table S1). These patients were classified as having either severe or mild lung disease, as defined by the lowest or highest quartile of forced expiratory volume in one second (FEV1), respectively, for age [7], [8].

### Determination of VNTR allele sizes

To estimate VNTR allele sizes for *MUC1*, *MUC2*, and *MUC5AC* genes, Southern blotting of *Hinf*I-digested genomic DNA was performed as described [18], with modifications to improve resolution. To minimize gel-to-gel variation in allele size identification, each gel included an internal DNA marker (mixture of DNA from 3 CEPH cell lines; Coriell Cell Repositories, Camden, New Jersey), which covered common *MUC1*, *MUC2* and *MUC5AC* VNTR alleles. *MUC7* VNTR alleles were examined by PCR.

### SNP genotyping

Genotyping for VNTR flanking SNPs was conducted by Applied Biosystems TaqMan® SNP Genotyping Assays, Illumina 610-Quad [8] or Illumina Golden Gate platforms.

### Statistical analysis

Differences in distributions of VNTR lengths between severe and mild CF patients were assessed by non-parametric Wilcoxon rank-sum tests (Intercooled Stata 10; College Station, TX). To define “short” versus “long” alleles in a non-biased manner for *MUC1* and *MUC5AC*, which both have a bimodal distribution, we estimated a finite mixture distribution using *mixdist* for the R computing environment. Fisher's exact or chi-square tests were used to test for association between “VNTR genotypes” and SNPs. Genetic associations between lung disease severity and SNPs were conducted using PLINK v1.07 (http://pngu.mgh.harvard.edu/purcell/plink/). Logistic regression, corrected for sex and ten principal components, was conducted for each cohort. HaploView version 4.2 was used to render LD maps. For two-variant haplotype analysis, each combination was tested by 100,000 permutations, and analyzed in R using *haplo.score*. To characterize the LD structure of SNPs associated with the 6.4 kb *MUC5AC* allele versus the 6.3 kb allele, the *haplo.em* module within the *haplo.stats* package was used (http://mayoresearch.mayo.edu/mayo/research/schaid_lab/software.cfm) [19]–[22].

## Results

### Allele distributions

**Figure 1:**
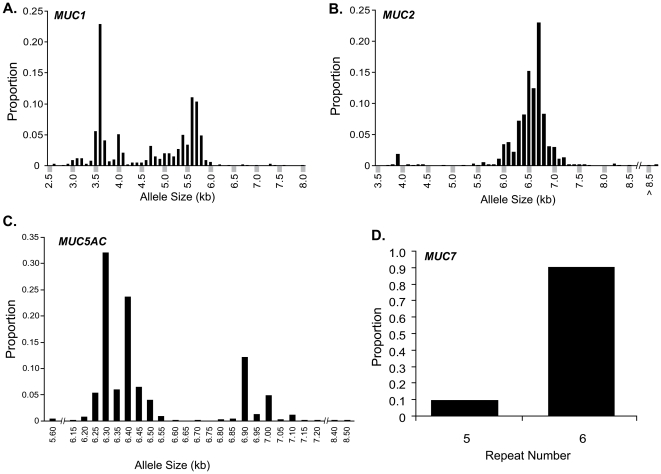
Distributions of VNTR allele sizes. Distributions of VNTR allele sizes as determined by Southern blot analysis of *Hinf*I-digested genomic DNA are shown for (A) *MUC1*, (B) *MUC2*, (C) *MUC5AC,* and by polymerase chain reaction for (D) *MUC7*.

Southern blots (*MUC1*, *MUC2*, *MUC5AC*) and PCR (*MUC7*) were used to estimate allele sizes (Figure S1), and allele distributions for VNTR length polymorphisms are shown (Figure 1). VNTR allele lengths were highly polymorphic. For *MUC1*, allele sizes ranged from 2.6 to 8 kb. The distribution was bimodal, with two relatively common peaks around 3.6 and 5.6 kb (Figure 1A). For *MUC2*, allele sizes ranged from 3.7 to >8.5 kb; however, the majority of alleles were between 5.9 to 7.2 kb, distributed around the mode at 6.7 kb (Figure 1B). For *MUC5AC*, alleles ranged from 5.6 to 8.5 kb, with a paucity of alleles <6.25 kb, >7.0 kb, and between 6.60 to 6.85 kb. Larger size alleles were relatively uncommon (Figure 1C). We observed two alleles for *MUC7* VNTRs, and the 5 repeat variant was infrequent (∼9%; Figure 1D).

### Association of allele sizes to lung phenotype

**Figure 2:**
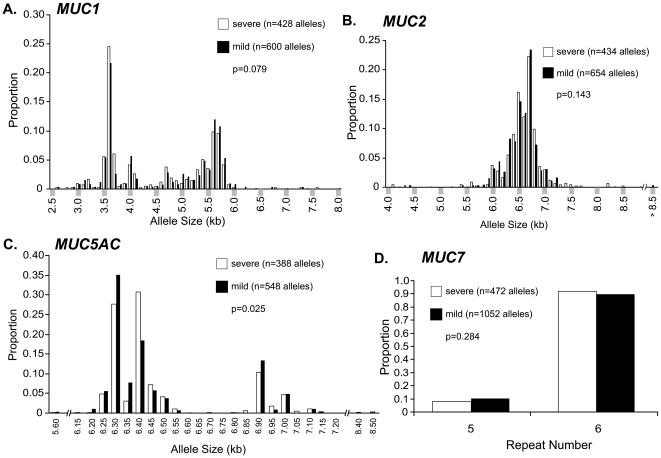
Distributions of VNTR allele sizes by lung severity status. Distributions of VNTR allele sizes by lung severity status for (A) *MUC1*, (B) *MUC2*, (C) *MUC5AC*, and (D) *MUC7* are shown. The number of chromosomes (alleles) representing each phenotype category for each mucin is given (n), as is the Wilcoxon rank-sum test p value.

We tested whether allele distribution was different between patients with severe versus mild CF lung disease (Figure 2). There was a trend toward significance for *MUC1* (p = 0.079; Figure 2A) but not for *MUC2* (p = 0.143; Figure 2B) or *MUC*7 (p = 0.284; Figure 2D). The *MUC5AC* association reached nominal statistical significance (p = 0.025; Figure 2C), although this did not survive correction for multiple comparison testing of 4 mucins (p = 0.1). For *MUC1*, the trend was for larger alleles (>4.9 kb) to be more common in mild CF patients. For *MUC5AC*, the difference in allele distribution was driven by 6.30/6.35 kb alleles being more prevalent in mild patients and 6.40/6.45 kb alleles more prevalent in severe patients (Figure 2C).

### Association by VNTR “genotype”

For *MUC1* and *MUC5AC* alleles, which had bimodal distributions (Figure 2A and 2C), we tested “short” (S) versus “long” (L) alleles for association with CF lung disease severity. For *MUC1*, because of the relatively continuous distribution of allele sizes, we initially used a statistical mixture fitting approach and defined a cut-point at 4.3 kb (S≤4.3 kb<L) (Figure S2A), but saw no association for allele-length genotypes and lung disease severity (p = 0.337; Figure S3A). However, because the statistically determined cut-point might not be most biologically relevant, we also analyzed short and long VNTR allele genotypes using a 4.9 kb cut-point, which is closer to the average value for the two most frequent alleles in the two modes of distribution, and which were used in previous studies [11]. This analysis showed a stronger trend for short alleles to be more common in severe patients, but did not reach significance (p = 0.059; Figure S3B). For *MUC5AC*, the statistical mixture-fitting approach defined the cut-point at 6.7 kb (S<6.7 kb≤L; Figure S2B), but no significant association of VNTR length genotypes with lung disease severity was seen (p = 0.914; Figure S3C).

We next considered the alternative hypothesis that individual VNTR alleles could associate with lung disease severity, either because they could contain functional variants, or act as proxies by LD with other genomic variants. Thus, we tested each VNTR allele individually for association with disease severity (only tested alleles with frequency ≥4%; Table 1 and Table S2). No significant association for *MUC2* alleles was found (Table S2). Nominal associations were found with the *MUC1* 3.7 kb and *MUC5AC* 6.35 and 6.30 kb alleles, but these did not survive correction for multiple testing (Table S2 and Table 1). In contrast, there was robust association between the *MUC5AC* 6.4 kb allele and severe lung disease, when the 6.4 kb allele was considered as the designated allele (D), versus all other alleles (p = 1.4×10^−5^). This strong association remained significant after Bonferroni correction for all 44 tests conducted (p = 6.2×10^−4^; Table 1).

**Table 1:** Association of *MUC5AC* VNTR allele sizes with lung disease severity (“Severe” versus “Mild”)†.

	D, D		D, non-D		non-D, non-D		Fisher's
	n (%)		n (%)		n (%)		Exact
Designated (“D”) Allele Size (kb)	Severe	Mild	Severe	Mild	Severe	Mild	P Value (nominal)
6.25	2 (1.0)	2 (0.7)	15 (7.7)	27 (9.9)	177 (91.3)	245 (89.4)	6.6×10^−1^
6.30	20 (10.3)	44 (16.1)	67 (34.5)	105 (38.3)	107 (55.2)	125 (45.6)	7.3×10^−2^
6.35	0 (0.0)	5 (1.8)	12 (6.2)	33 (12.1)	182 (93.8)	236 (86.1)	1.2×10^−2^ ‡
6.40	22 (11.3)	21 (7.7)	76 (39.2)	59 (21.5)	96 (49.5)	194 (70.8)	1.4×10^−5^ *
6.45	1 (0.5)	1 (0.4)	27 (13.9)	29 (10.6)	166 (85.6)	244 (89.0)	4.8×10^−1^
6.50	4 (2.1)	4 (1.5)	8 (4.1)	13 (4.7)	182 (93.8)	257 (93.8)	8.6×10^−1^
6.90	4 (2.1)	5 (1.8)	32 (16.5)	64 (23.4)	158 (81.4)	205 (74.8)	1.9×10^−1^
7.00	0 (0.0)	4 (1.5)	18 (9.3)	19 (6.9)	176 (90.7)	251 (91.6)	1.7×10^−1^

For each test, a specific size is denoted the “Designated” allele (allele D), and is compared to all other alleles (non-D). The number of patients (n = 468; Severe = 194; Mild = 274) with each genotype (D, D; D, non-D; and non-D, non-D) is given for each test.

† Analyzed only for allele sizes that were present in ≥ 4% of the population.

‡ 6.35 kb (and 6.30 kb) allele tends to associate with “mild” disease, but is not significant after Bonferroni correction.

*6.4 kb allele associates with “severe” lung disease; Bonferroni corrected p value for the tests indicated in this Table (8 tests total), p = 1.1×10^−4^; corrected p value for the tests in this Table, plus the original tests for association with lung disease severity shown in Figure 2 (12 tests total), p = 1.7×10^−4^; corrected p value for all tests directly above, plus the additional tests utilizing the cut-point analysis in Figure S3 (15 tests total), p = 2.1×10^−4^; corrected p value for all tests directly above, plus the *MUC1* and *MUC2* VNTR allele size tests for association (data not shown; 29 tests total), p = 4.1×10^−4^; corrected p value for all tests directly above, plus SNPs in Table S3 (44 tests total), p = 6.2×10^−4^.

### SNP associations

Analysis of SNP data from candidate gene studies of *MUC1, MUC2*, and *MUC5AC* showed that none of the genotyped SNPs were highly associated with lung disease severity (Table S3). The strongest association was with rs10902076, upstream of *MUC2* (p = 4.5×10^−3^), but the association did not survive correcting for multiple comparisons (p = 0.068; Table S3), and replication studies are needed to support this finding. Additionally, the larger GWAS showed no significant associations with SNPs close to these mucin genes [8].

### Association of SNPs to VNTR allele sizes

We next characterized LD patterns of mucin VNTRs with nearby SNPs. SNPs in high LD with VNTRs might be useful proxies for VNTR alleles; plus LD structures would be useful for other investigations involving mucin genes, since VNTR regions cannot be adequately queried on current genotyping platforms. Available *MUC2* SNPs showed weak LD to *MUC2* VNTR sizes, whereas there was moderately strong LD between SNPs and VNTRs for *MUC7*, similar to previously reported [15], [23].

**Figure 3:**
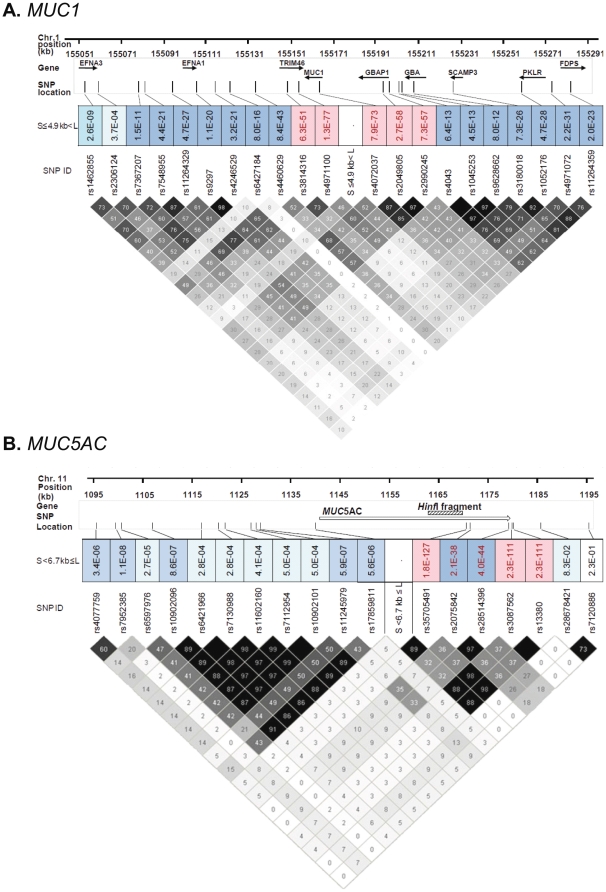
Schematic representations of *MUC1* and *MUC5AC* LD structure. The LD structure is shown for (A) *MUC1* (n = 514 patients; human genome build 19) and (B) *MUC5AC* genes (n = 468 patients; human genome build 18 was used due to incomplete build information in this region in build 19). The location of the SNPs (minor allele frequency >9%) that have been evaluated are shown, along with their relative chromosome position. The LD structure is shown, considering cut-points to assign genotypes for *MUC1* (cut-point 4.9 kb; short≤4.9 kb<long) and *MUC*5*AC* (cut-point 6.7 kb; short<6.7 kb≤long). The plot was constructed with the Haploview program and the pairwise *r* ^2^ (×100) values are depicted in the diamonds. The dark diamonds have higher *r* ^2^ ((×100) values, and the diamonds in lighter shade of color have lower *r* ^2^ ((×100) values. The colored boxes above the LD map represent the p value from chi-square tests to evaluate the significance of the SNP genotype to the VNTR allele genotypes. Color coding: white (*p*>0.1), light blue (10^−5^<p≤0.1); medium blue (10^−10^<p≤10^−5^); dark blue (10^−50^<p≤10^−10^) and red (p≤10^−50^).

For *MUC1* and *MUC5AC*, we tested for LD between flanking SNPs and genotypes for VNTR allele genotypes, as defined above, and the locations of SNPs (minor allele frequency >9%) and LD status are shown (Figure 3).

***MUC1*** **.** There was strong LD between flanking SNPs and defined VNTR genotypes (S≤4.9 kb<L), extending in both directions from the VNTR. The strongest LD with VNTRs was in the gene region between rs3814316 and rs2990245 (Figure 3A).

***MUC5AC*** **.** The *MUC5AC* LD analysis was similar to *MUC1* in that strong LD was discovered between flanking SNPs and VNTR genotypes (cut-point S<6.7 kb≤L) (Figure 3B). However, unlike *MUC1,* where very strong LD was found on both sides of the VNTR region, LD for *MUC5AC* was strongest on the 3′ side, in the gene region between rs35705491 and rs13380. Within this LD block, rs2075842 and rs28514396 are in near perfect LD, but exhibit reduced LD to SNPs flanking either side (Figure 3B). These SNPs are important in subsequent analyses to distinguish VNTR alleles. SNPs between rs10902096 and rs17859811 (except rs11245979) form a strong LD block, but this block is not in significant LD with the VNTR S<6.7 kb≤L genotypes.

**Figure 4:**
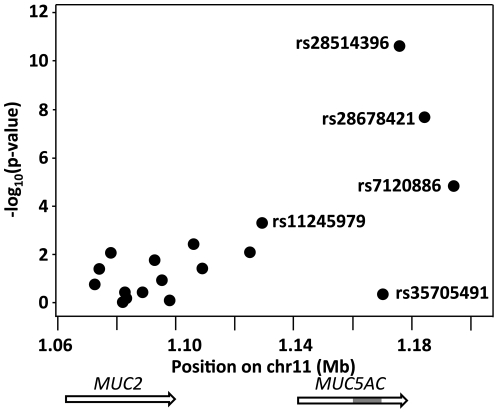
Analysis of differential linkage disequilibrium between *MUC5AC* 6.3 and 6.4 kb alleles versus nearby SNPs. P values represent evidence of an improved fit to the LD patterns between SNPs and the VNTR when 6.3/6.4 are treated as separate VNTR alleles (three-allele system) instead of being collapsed into a single allele (two-allele system). The p values represent a direct test of the hypothesis that the 6.3 kb and 6.4 kb alleles follow distinct LD patterns with the surrounding SNPs. Non-significant p values, e.g., those 5′ to *MUC5AC*, indicate that the two alleles share the same LD structure with the SNPs, while significant p values indicate that the LD pattern between the two alleles is different, e.g., at rs28514396. Approximate location of *Hinf*I fragment in *MUC5AC* shown in gray.

Since the 6.4 kb allele of *MUC5AC* was associated with severe CF lung disease, we next tested whether the severity-associated 6.4 kb allele could be distinguished from other alleles (especially the 6.3 kb allele, which is similar in size and in frequency), based upon LD patterns with nearby SNPs. In order to simplify the comparisons for testing, we considered an artificial three-allele system with VNTR alleles 6.3 kb and 6.4 kb treated separately, and all other VNTR alleles collapsed into a single allele (Text S1). We estimated the LD pattern of this three-allele VNTR with each of the flanking SNPs, and then compared the strength of this association to that observed when the VNTR was classified according to an artificial two-allele system (6.3 kb/6.4 kb combined into one allele versus all others). Likelihood ratio testing of the VNTR three-allele system versus the two-allele system showed highly significant evidence of improved fits from the three-allele system (Figure 4), indicating that LD structure with nearby SNPs was better explained by considering the 6.3 and 6.4 kb alleles as separate alleles, rather than as the same allele (as in Figure 3, where both alleles are grouped as “short”). For many SNPs in the region, the two alleles do act similarly (as indicated by non-significant P-values in Figure 4); however, for other SNPs (rs28514396; rs28678421; rs7120886), the pattern of association of the 6.4 kb and 6.3 kb allele is clearly different (p = 2.03×10^−11^ for rs28514396). This striking result easily meets multiple comparison correction for the SNPs examined. In this manner, we provided a rigorous test of a genetically meaningful difference between the 6.3 kb and 6.4 kb alleles, not only providing direct evidence of the accuracy of the sizing on the Southern blot, but also providing further evidence that the association of the 6.4 kb allele with CF lung disease severity is deserving of careful follow-up.

Given these findings, we hypothesized that the phenotypic association seen with the *MUC5AC* 6.4 kb allele could be represented by extended haplotypes that included SNPs. We inferred haplotypes and tested association for two-variant haplotypes [22]. Haplotypes containing the 6.4 kb VNTR allele had strong associations with severe CF lung disease, but haplotypes that did not include the 6.4 kb allele, including those that contained only SNPs, were not significant (Table S4). As expected from the distribution analyses (Figure 2C), haplotypes containing the 6.3 kb VNTR allele tended to be associated with mild disease, but the associations were not robust. We conclude that even though the 6.4 kb allele can be distinguished from other VNTR alleles (Figure 4), the SNPs thus far tested do not clearly tag the 6.4 kb severity-associated variant (Table S4).

## Discussion

To evaluate the hypothesis that mucin gene VNTR polymorphisms are associated with severity of CF lung disease, we used *Hinf*I-digested DNA and Southern blot to determine the VNTR allele length polymorphisms for *MUC1, MUC2,* and *MUC5AC*, and used PCR methods for *MUC7*. Southern blot experiments were carefully optimized to maximize the reliability of allele size calls, using a mixture of samples from patients with “severe” and “mild” lung disease on each gel, standard internal markers and size standards, and a genetically homogenous CF population (Caucasian Phe508del homozygotes). Coupled with the availability of SNP genotypes from larger studies [8], this current report represents the largest and most comprehensive evaluation of mucin VNTR regions available to date, and the first of its kind in CF patients.

Our results confirm the polymorphic nature of VNTR regions, as previously described [10], [12], [14], [17]. The *MUC1* allele distribution in our population was similar to that previously reported in Europeans [12], but differs from that reported in Japanese [10], who have fewer large size alleles and less distinct bimodal distribution. The distributions of allele sizes, in our patients for *MUC2*, *MUC5AC*, and *MUC7*, is similar to published work [14], [17]; however, our data provide more fine detail. For example, although both *MUC1* and *MUC5AC* appear to have a “bimodal” distribution as reported previously, we demonstrated small “peaks” at 4.0 and 4.7 kb for *MUC1* (Figure 1A) and large clusters of alleles at 6.3 and 6.4 kb for *MUC5AC* (Figure 1C).

We initially hypothesized that the core VNTR length would correlate with disease severity. While there was a trend toward significance for *MUC1* VNTR distribution to be different between severe and mild patients, only the *MUC5AC* association reached statistical significance (Figure 2C). However, when *MUC5AC* VNTR alleles were classified into “genotypes” by size (short or long alleles: S or L, respectively), there was not significant association with disease severity by genotype (Figure S3C). Thus, our initial hypothesis that VNTR size would be directly correlated to phenotype was not confirmed. However, when we tested the alternative hypothesis, i.e., that individual VNTR size variants could associate with lung disease, we discovered a robust association between the *MUC5AC* 6.4 kb VNTR allele and severe lung disease (p = 6.2×10^−4^ after Bonferroni correction; Table 1).

The availability of SNP genotype data in flanking regions provided the opportunity to evaluate LD of SNPs with VNTR size alleles. For both *MUC1* and *MUC5AC*, there was strong association between the VNTR genotype (short and long alleles; Figure S2) and flanking SNPs (Figure 3), which is consistent with previous results in smaller patient populations [24], [25]. The SNPs in strong LD could possibly be used as proxies for short/long VNTR alleles, which may be useful for interpreting other GWAS studies, if associations to *MUC1* and *MUC5AC* SNPs are seen.

Flanking SNPs were also used to formally demonstrate that the *MUC5AC* 6.4 kb allele is genetically distinct from other VNTR alleles, particularly the 6.3 kb allele (Figure 4). The demonstration that the 6.4 kb allele is genetically distinct ruled out technical issues related to Southern blot resolution, and will enable future efforts to define functional variants associated with the 6.4 kb allele. None of the tested SNPs were able to strongly tag the causal allele, and we were unable to define any SNP haplotypes that show the same phenotypic association as the 6.4 kb allele. The genomic structure of *MUC5AC* is poorly mapped and assembled in public databases, and our lack of SNP association likely reflects the paucity of pertinent SNPs available for genotyping. While the possibility exists that the 6.4 kb allele association is due to a type 1 error (false positive), our findings support the concept that the 6.4 kb *MUC5AC* allele either contains or is linked to important functional variants, some of which may be involved in diverse functions outside those related to VNTR variants.

Functional variants in mucin genes are very likely in humans, as polymorphisms within the amino acid motifs of the VNTR regions are common [26], [27], and alternative splicing also creates potentially functional variations [28]. Amino-acid substitutions in other regions may also affect function, for example, this has been seen in mutant mice with colitis, where specific amino-acid changes prevent proper processing and secretion [29].

To our knowledge, this is the first published association of *MUC5AC* VNTR alleles with lung disease phenotypes. The odds ratio for 1 or 2 copies of the 6.4 kb *MUC5AC* VNTR allele to be associated with severe CF lung disease is 2.5. Extrapolating from methods recently published [8], [30], this translates to a difference in the CF quantitative lung phenotype of 0.3 units, which equates to an average difference of 7.7% in FEV1 (% predicted) between adult patients that do not carry the 6.4 kb allele (n = 290) versus those patients that carry one or two copies (n = 178). This is a substantial adverse effect, resulting in an average difference in raw FEV1 of ∼275 ml, which is similar to values calculated for the most significant association in the recently published GWAS study, which queried a much larger CF population [8].

MUC5AC is a major mucin in airway secretions of healthy subjects, and it is often up-regulated under a variety of pathogenic conditions, including CF, COPD, smoking, and asthma [31]–[34]. The specific roles of MUC5AC in the normal lung, apart from other secreted mucins, have not yet been fully defined, but it is reasonable to speculate that it is involved in innate defense mechanisms associated with mucociliary clearance. During disease, when innate defense mechanisms are altered or fail, the presence of the correct levels of MUC5AC of appropriate structure may be critically important for airway defense. In a mouse model, Muc5ac was recently shown to be a critical component in the immune regulated rejection of enteric nematodes [35]. *MUC5AC* VNTR length variants (which may confer differing structural properties), could alter the function of the airway mucus, affecting the inflammation/defense status and the progression of lung disease [1], [2], [16]. Alternatively, and/or additionally, the 6.4 kb allele may be associated with some unknown genetic variants in the inaccessible repetitive *MUC5AC* regions or other parts of the coding region, or with regulatory variants that affect expression, as suggested for a polymorphism in *MUC5B*, in a recent publication [36]. Whatever is the case, the alleles described here might be relevant for other serious lung diseases, and further studies to define the mechanistic link between the *MUC5AC* VNTR functional variants and CF lung disease are warranted.

## Supporting Information

Figure S1 **Representative Southern blots of** ***HinfI*** **-digested genomic DNA.** DNA was probed for *MUC1* (A), *MUC2* (B), and *MUC5AC* (C). PCR analysis of the *MUC7* (D) repeat polymorphism is shown. The *MUC7* genotypes represent either 5 or 6 repeats of the VNTR. Heterozygotes have an additional hetero-duplex band. M = molecular weight marker; M3 = mixture of CEPH DNA as described in Methods and Text S1.
(TIF)

Figure S2 **Mixture fitting to define cut-points for short/long alleles for** ***MUC1*** **(A) and** ***MUC5AC*** **(B).** The statistically selected cut-points for each mucin are shown with the black arrows, and they were taken as the average of two standard deviations from the mean of the respective distributions. The green lines represent the distribution trend with the two peaks illustrating a bimodal mode. The blue lines represent the distribution of the alleles. The red lines show all alleles calculated with red arrows indicating the summit of each peak.
(TIF)

Figure S3 **Genotype distribution by severity status for VNTR length as defined by cut-points.** The genotype distribution by severity status for VNTR length is shown for *MUC1* (A) and *MUC5AC* (C), as defined by cut-point (see Figure S2). Genotype distribution by severity status, based upon the previously published defined cut-points (“biological”), is shown for *MUC1* (B). The number (n) of severe and mild patients is provided for each analysis, as are the nominal p values (Fisher exact test).
(TIF)

Text S1 **Supporting information text for Methods.**
(DOC)

Table S1 **Characteristics of CF patients used for VNTR analysis.**
(DOC)

Table S2 **Association of** ***MUC1*** **and** ***MUC2*** **VNTR allele sizes with lung disease severity.**
(DOC)

Table S3 **Association of** ***MUC1*** **,** ***MUC2*** **and** ***MUC5AC*** **SNPs with CF lung phenotype.**
(DOC)

Table S4 **Two-variant haplotype analysis for 6.3 and 6.4 kb** ***MUC5AC*** **VNTR alleles with flanking SNPs.**
(DOC)
