# Supplementary figures and images for: Mucin Variable Number Tandem Repeat Polymorphisms and Severity of Cystic Fibrosis Lung Disease: Significant Association with MUC5AC
Source: PLoS One. 2011 Oct 6;6(10):e25452. doi: 10.1371/journal.pone.0025452 (PMC3188583; doi:10.1371/journal.pone.0025452)

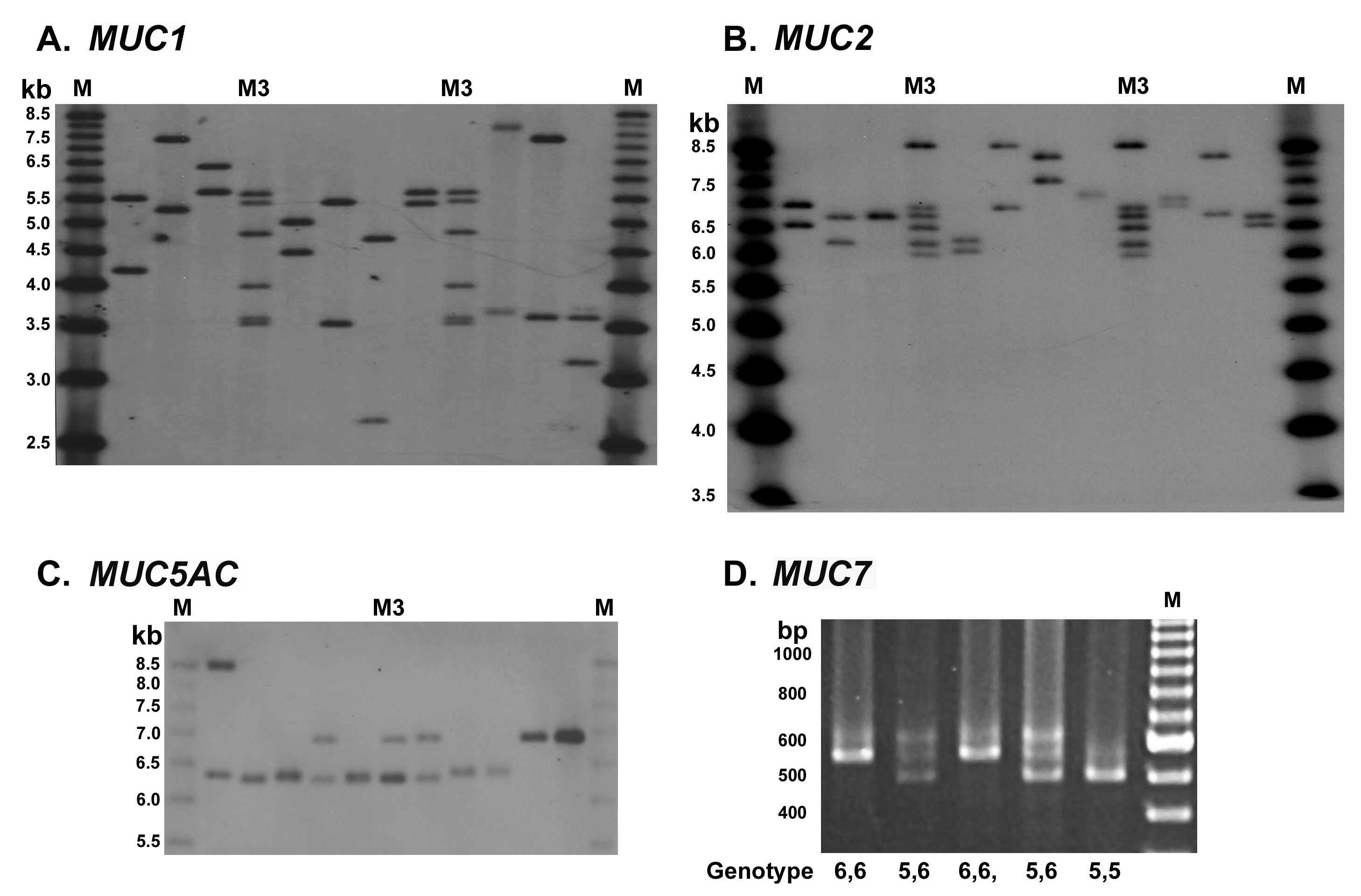

Supplement: Figure S1 — Representative Southern blots of HinfI -digested genomic DNA. DNA was probed for MUC1 (A), MUC2 (B), and MUC5AC (C). PCR analysis of the MUC7 (D) repeat polymorphism is shown. The MUC7 genotypes represent either 5 or 6 repeats of the VNTR. Heterozygotes have an additional hetero-duplex band. M = molecular weight marker; M3 = mixture of CEPH DNA as described in Methods and Text S1. (TIF) [file pone.0025452.s001.tif]

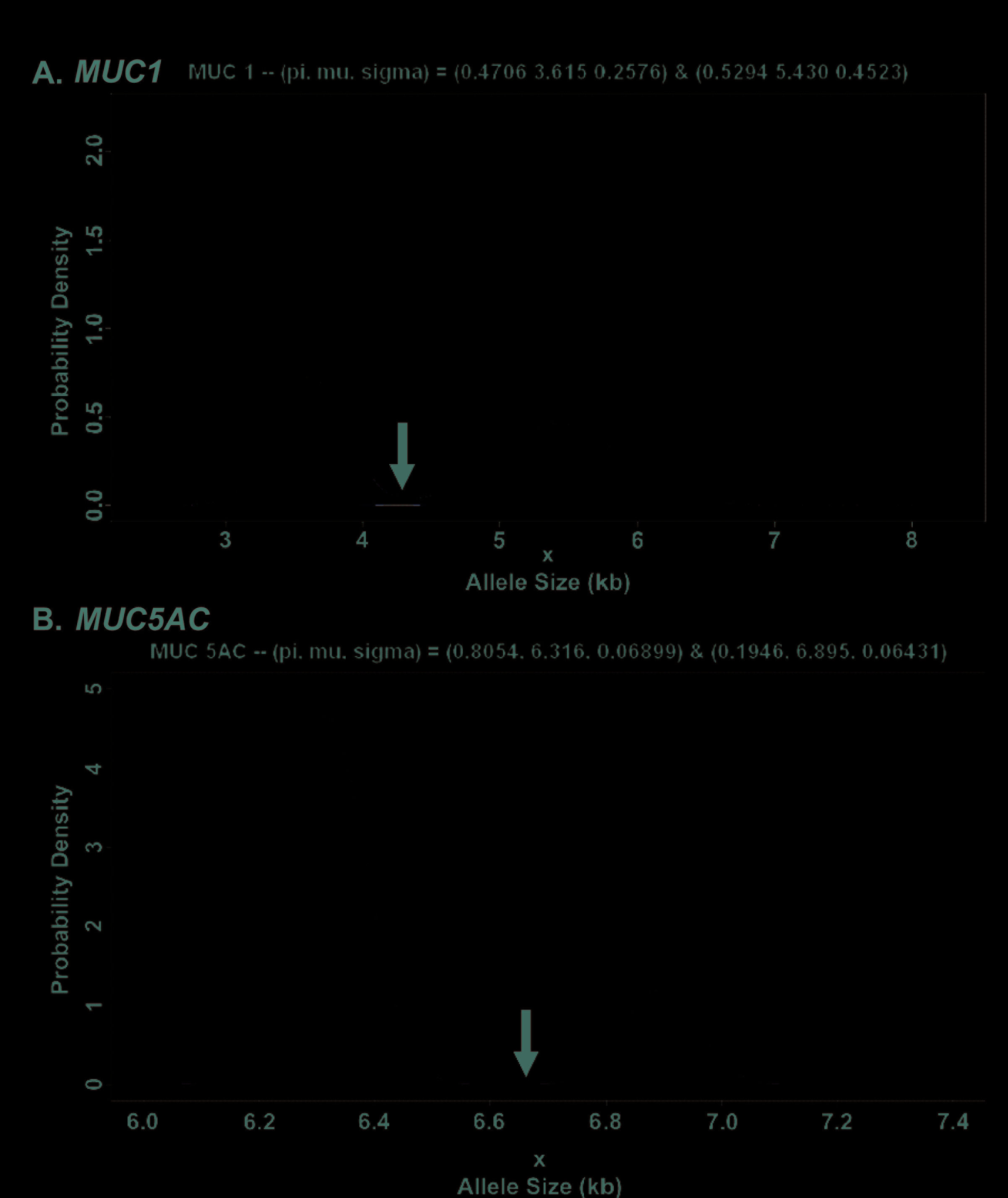

Supplement: Figure S2 — Mixture fitting to define cut-points for short/long alleles for MUC1 (A) and MUC5AC (B). The statistically selected cut-points for each mucin are shown with the black arrows, and they were taken as the average of two standard deviations from the mean of the respective distributions. The green lines represent the distribution trend with the two peaks illustrating a bimodal mode. The blue lines represent the distribution of the alleles. The red lines show all alleles calculated with red arrows indicating the summit of each peak. (TIF) [file pone.0025452.s002.tif]

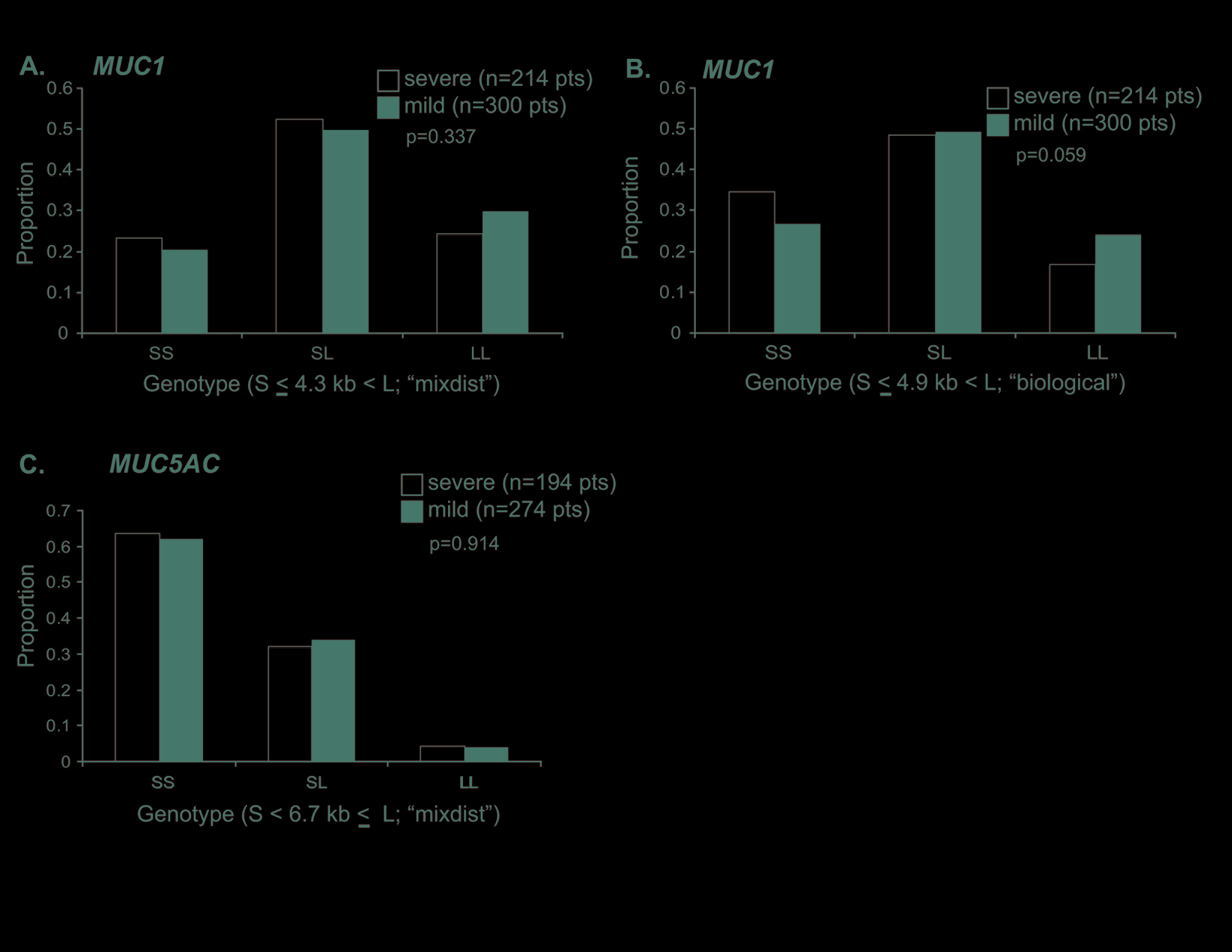

Supplement: Figure S3 — Genotype distribution by severity status for VNTR length as defined by cut-points. The genotype distribution by severity status for VNTR length is shown for MUC1 (A) and MUC5AC (C), as defined by cut-point (see Figure S2). Genotype distribution by severity status, based upon the previously published defined cut-points (“biological”), is shown for MUC1 (B). The number (n) of severe and mild patients is provided for each analysis, as are the nominal p values (Fisher exact test). (TIF) [file pone.0025452.s003.tif]
