# Supplementary material for: Mucin Variable Number Tandem Repeat Polymorphisms and Severity of Cystic Fibrosis Lung Disease: Significant Association with MUC5AC
Source: PLoS One. 2011 Oct 6;6(10):e25452. doi: 10.1371/journal.pone.0025452 (PMC3188583; doi:10.1371/journal.pone.0025452)
Supplement: Text S1 — Supporting information text for Methods. (DOC) [file pone.0025452.s004.doc]

**Supporting Information Text**

**Mucin Variable Number Tandem Repeat Polymorphisms and Severity of Cystic Fibrosis Lung Disease: Significant Association with MUC5AC**

**Methods**

**Determination of VNTR allele sizes:** Patients for the VNTR analyses were selected for availability of sufficient, high quality DNA [1, 2]. For the major aspects of this work, digested DNA (2 μg) was fractionated by electrophoresis in 0.7 % (w/v) agarose (Sigma-Aldrich, St. Louis, MO) gel in 1X TBE buffer. The gels were depurinated in 0.25 M HCl for 30 mins, denatured for 40 mins in 1.5M NaCl/0.5M NaOH, neutralized in 0.5 M Tris HCl/0.001M EDTA, pH 7.2 for 40 mins, and then transferred to positively charged nylon membranes (Roche, Indianapolis, IN) by capillary transfer for 18 hrs.

Agarose gels for the *Hinf*I fragments were electrophoresed under conditions optimized to maximize resolution between the sizes of 3.0 and 8.5 kb. Southern blots were probed with mucin-specific tandem repeat probes and were visualized with DIG detection system (Roche, Indianapolis, IN). Generally, the digested *Hinf*I fragments were run at 4°C for 26.5 hours under constant voltage (2.35 V/cm), which allowed for successful identification of *MUC2* and *MUC5AC* VNTR alleles and a large percentage of *MUC1* alleles. Additional gels were run to maintain fragments less than 3 kb on selected patients that potentially carried a smaller *MUC1* allele (patients not heterozygous for larger alleles). Subsequent gels were also run to increase resolution on eighty patients having 1 or 2 of the common *MUC5AC* alleles (6.30 kb to 6.45 kb) by increasing the gel run time by 16 hours at 2 V/cm after a complete change of the tank buffer.

Southern blot membranes were probed with the following probes:

*MUC1* oligo probe: 5'CTCCACCGCCCCCCCAGCCCACGGTGTCACCTCGGCCCCGGACACCAGGCCGGCCCCGGG

*MUC2* oligo probe: 5'CCAACCACGACACCCATCACCACCACCACTACGGTGACCCCAACCCCAACACCCACCGGCACACAGACC

*MUC5AC* oligo probe:

5’CCTACAACCAGCACAACCTCTGCTCCTACAACCAGCACAACCTCTGCT

All oligo probes were obtained from Eurofins MWG Operon (Huntsville, AL). Blots were stripped between probings in a solution of 0.2M NaOH/0.1% SDS, hybridized at 42°C for 16 hours in Easy Hyb solution (Roche), and labeled with the 3' DIG labeling kit (Roche).

Original data on Centre d'Etude du Polymorphisme Humain (CEPH) (commercially available from Coriell Cell Repositories, Camden, New Jersey) cell line allele sizes were kindly provided by D.M. Swallow. Specifically, we used cell line ID numbers GM12275, GM12663, and GM12672, for internal markers to provide landmarks for the gels. In addition, each gel contained 3 lanes of 10 μg of a 500 bp DNA ladder (500-8500 bp ladder; Invitrogen, Carlsbad, CA) that was labeled as described above. All ladders, samples, and internal markers had the same final volume and salt concentration (18.33 mM) when loaded onto the gel. Allele sizes were read manually using a 500 bp ladder and the internal markers to provide landmarks. Gels were scored with the investigator blinded to sample disease status.

The number of patients studied varied among mucin genes. For *MUC7*, which was a PCR based assay, additional patients were run. Patient data were excluded if the Southern blot was not considered reliable (due to low quality DNA) for a specific mucin. Additionally, some alleles critical to data interpretation were confirmed on additional gels and double-checked by another molecular biology expert (W.K.O. or M.R.K.).

**PCR for *MUC7* VNTR allele:** The *MUC7* VNTR allele polymorphism was examined by PCR, using primers designed to amplify the VNTR region such that an allele containing 5 or 6 tandem repeats produced a product of 505 and 574 bp, respectively. The *MUC7* PCR primers were as follows:

forward, 5'ATGCCACCACCATATCTTCAAG

reverse, 5'GAAGTTTCAGAAGTGTCAGGTGC

PCR for *MUC7* was performed in a 25 μl reaction volume containing 100 ng genomic DNA, 0.4 μM each forward and reverse primer, 1X buffer [1mM (NH_4_)^2^SO_4_, 67mM Tris-HCl (pH8.8), 0.01% Tween-20], 1-3 mM MgCl_2_, 100-200 μM dNTP mix, and 0.8 units of REDTaq® DNA polymerase (Sigma-Aldrich, St. Louis, MO). PCR amplification was carried out using the GeneAmp 9700 ThermoCycler (Applied Biosystem, Foster City, CA). Reaction conditions were as follows:  5 min denaturation at 94°C; 37 cycles of 94°C for 30 sec, 64°C for 35 sec, 72°C for 55 sec; and one final extension of 10 min at 72°C. The PCR products were subjected to electrophoresis on 2% agarose gels at RT at 4.5 V/cm for 45-50 minutes and visualized with ethidium bromide staining under UV light.

**PCR to Genotype SNPs:** Standard protocol was used to generate SNP data by either TaqMan® SNP Genotyping Assays (run on ABI 7500 Real-Time PCR Systems instrument) or Illumina 610 Quad or Illumina GoldenGate platforms.

**Statistical Analysis:** The cut-point differentiating the short and long VNTR alleles for *MUC1* and *MUC5AC* using *mixdist*, was taken as the average of two standard deviations from the mean of the respective distributions [3]. Logistic regression, corrected for sex and ten principal components {obtained from SMARTPCA using a thinned set of ~93,000 markers, as listed in [4]}, was carried out for each cohort in whom VNTR size alleles were available.

**Analysis of differential linkage disequilibrium (LD) between the *MUC5AC* 6.3 kb and 6.4 kb alleles versus nearby SNPs:** The hypothesis to be tested is whether the 6.3 kb and 6.4 kb VNTR alleles (hereafter simply "6.3" and "6.4") are distinct in terms of their LD relationship with nearby SNPs. Precisely, the null hypothesis is that the 6.3 and 6.4 have identical LD patterns with each of the SNPs in the region, but, in order for the hypothesis to be accurately tested, the statistical analysis must also take into account the differing frequencies of the two alleles in the population. In haplotype frequency terms, the null hypothesis is that for any nearby SNP with alleles A and B, the ratio of haplotype frequencies *freq*(6.3--A)/*freq*(6.4--A) is equal to the individual marginal frequencies of 6.3 to 6.4. The same ratio applies for SNP allele B.  In other words, while there may be strong evidence of LD between the VNTR and the SNP, the null hypothesis is that there is no *differential* LD with the SNP when comparing the 6.3 VNTR allele to the 6.4 allele.

In order to test the hypothesis, for each SNP near *MUC5AC* we used the *haplo.em* module of the *haplo.stats* v. 1.4.4 R package (<http://mayoresearch.mayo.edu/mayo/research/schaid_lab/software.cfm>) to fit the maximum likelihood pattern for the VNTR vs. the SNP [5]. For the “full” VNTR model, we considered the three-allele system {6.3, 6.4, other}, while for the “reduced” model we considered the two-allele VNTR system {6.x, other}, where 6.x stands for either the 6.3 or 6.4 allele. Obtaining these maximum likelihood values in the appropriate nested ratio requires computing an appropriate offset to account for differing frequencies of heterozygotes (Dr. Daniel Schaid, personal communication). The resulting 2X(log-likelihood ratio) is approximately distributed as χ_2_^2^ under the null. The test was then performed separately for each SNP in the region, resulting in the plot shown in Figure 4 of the main manuscript.

**REFERENCES**

1. Drumm ML, Konstan MW, Schluchter MD, Handler A, Pace R, et al. (2005) Gene modifiers of lung disease in cystic fibrosis. N Engl J Med 353:1443-1453.

2. Wright FA, Strug LJ, Doshi VK, Commander CW, Blackman SM, et al. (2011) Genome-wide association and linkage identify modifier loci of lung disease severity in cystic fibrosis at 11p13 and 20q13.2. Nat Genet 43(6):539-546.

3. Du, J (2002) Combined Algorithms for Fitting Finite Mixture Distributions. McMaster University.

4. Taylor C, Commander CW, Collaco JM, Strug LJ, Li W, et al. (2011) A novel lung disease phenotype adjusted for mortality attrition for cystic fibrosis genetic modifier studies. Pediatr Pulmonol 46(9):857-869.

5. Schaid DJ, Rowland CM, Tines DE, Jacobson RM, Poland GA (2002) Score tests for association between traits and haplotypes when linkage phase is ambiguous. Am J Hum Genet 70:425-434.
