# Supplementary material for: Mucin Variable Number Tandem Repeat Polymorphisms and Severity of Cystic Fibrosis Lung Disease: Significant Association with MUC5AC
Source: PLoS One. 2011 Oct 6;6(10):e25452. doi: 10.1371/journal.pone.0025452 (PMC3188583; doi:10.1371/journal.pone.0025452)
Supplement: Table S1 — Characteristics of CF patients used for VNTR analysis. (DOC) [file pone.0025452.s005.doc]

**Table S1.** **Characteristics of CF patients used for VNTR analysis.**

| **Mucin Gene** | ***MUC1*** | | ***MUC2*** | | ***MUC5AC*** | | ***MUC7*** | |
| --- | --- | --- | --- | --- | --- | --- | --- | --- |
| **CF Lung Disease Severity** | **Severe** | **Mild** | **Severe** | **Mild** | **Severe** | **Mild** | **Severe** | **Mild** |
| **Number of pts**† | 214 | 300 | 217 | 327 | 194 | 274 | 236 | 526 |
| **Age* (yrs) range** | 8 - 25 | 15 - 52 | 8 - 25 | 15 - 52 | 8 - 25 | 15 - 52 | 8 - 25 | 15 - 54 |
| **Age* (yrs) mean ± SD** | 16.3 ± 4.1 | 28.1 ± 9.7 | 16.4 ± 4.1 | 28.0 ± 9.7 | 16.3 ± 4.2 | 28.0 ± 9.8 | 16.5 ± 4.0 | 28.8 ± 9.8 |
| **Male; n (%)** | 110 (51%) | 159 (53%) | 106 (49%) | 177 (54%) | 100 (52%) | 149 (54%) | 117 (50%) | 290 (55%) |
| **FEV_1_* (% predicted) mean ± SD** | 46.7 ± 17.6 | 74.3 ± 29.0 | 46.4 ± 17.4 | 74.6 ± 28.7 | 46.4 ± 17.3 | 74.5 ± 28.7 | 45.9 ± 16.6 | 72.9 ± 28.9 |

† All patients are Caucasian and homozygous for the Phe508del *CFTR* mutation, and patient numbers vary due to Southern blot quality differences (due to low quality DNA), which eliminated some patients from each group

* At time of enrollment into study
