# Supplementary material for: Mucin Variable Number Tandem Repeat Polymorphisms and Severity of Cystic Fibrosis Lung Disease: Significant Association with MUC5AC
Source: PLoS One. 2011 Oct 6;6(10):e25452. doi: 10.1371/journal.pone.0025452 (PMC3188583; doi:10.1371/journal.pone.0025452)
Supplement: Table S2 — Association of MUC1 and MUC2 VNTR allele sizes with lung disease severity. (DOC) [file pone.0025452.s006.doc]

**Table S2.** **Association of *MUC1* and *MUC2* VNTR allele sizes with lung disease severity^†^.**

|  | **D, D** | | **D, non-D** | | **non-D, non-D** | | **Fisher's Exact** |
| --- | --- | --- | --- | --- | --- | --- | --- |
| **Designated “D”** | **n (%)** | | **n (%)** | | **n (%)** | | **P Value** |
| **Allele Size (kb)** | **Severe** | **Mild** | **Severe** | **Mild** | **Severe** | **Mild** | **(nominal)** |
| ***MUC*1** | | | | | | | |
| 3.5 | 0 (0.0) | 0 (0.0) | 24 (11.2) | 33 (11) | 190 (88.8) | 267 (89.0) | 1 |
| 3.6 | 10 (4.7) | 11 (3.7) | 85 (39.7) | 108 (36.0) | 119 (55.6) | 181 (60.3) | 5.3 x 10^-1^ |
| 3.7 | 1 (0.5) | 1 (0.3) | 24 (11.2) | 14 (4.7) | 189 (88.3) | 285 (95.0) | 9.0 x 10^-3^ * |
| 4.0 | 0 (0.0) | 1 (0.3) | 18 (8.4) | 32 (10.7) | 196 (91.6) | 267 (89.0) | 6.3 x 10^-1^ |
| 5.4 | 0 (0.0) | 1 (0.3) | 22 (10.3) | 27 (9.0) | 192 (89.7) | 272 (90.7) | 8.0 x 10^-1^ |
| 5.6 | 1 (0.5) | 6 (2.0) | 40 (18.7) | 60 (20.0) | 173 (80.8) | 234 (78.0) | 3.6 x 10^-1^ |
| 5.7 | 3 (1.4) | 5 (1.7) | 35 (16.4) | 55 (18.3) | 176 (82.2) | 240 (80.0) | 8.3 x 10^-1^ |
| 5.8 | 0 (0.0) | 2 (0.7) | 18 (8.4) | 28 (9.3) | 196 (91.6) | 270 (90.0) | 6.0 x 10^-1^ |
| ***MUC*2** | | | | | | | |
| 6.3 | 2 (0.9) | 5 (1.5) | 20 (9.2) | 44 (13.5) | 195 (89.9) | 278 (85.0) | 2.7 x 10^-1^ |
| 6.4 | 6 (2.8) | 5 (1.5) | 28 (12.9) | 40 (12.2) | 183 (84.3) | 282 (86.3) | 5.7 x 10^-1^ |
| 6.5 | 12 (5.5) | 12 (3.7) | 46 (21.2) | 72(22.0) | 159 (73.3) | 243 (74.3) | 6.1 x 10^-1^ |
| 6.6 | 7 (3.2) | 12 (3.7) | 38 (17.5) | 59 (18.0) | 172 (79.3) | 256 (78.3) | 9.5 x 10^-1^ |
| 6.7 | 20 (9.2) | 33 (10.1) | 56 (25.8) | 88 (26.9) | 141 (65.0) | 206 (63.0) | 9.0 x 10^-1^ |
| 6.8 | 3 (1.4) | 4 (1.2) | 37 (17.0) | 40 (12.2) | 177 (81.6) | 283 (86.6) | 2.8 x 10^-1^ |

For each test, a specific size is denoted the “Designated” allele (allele D), and is compared to all other alleles (non-D) in severe and mild groups. The number of patients with each genotype (D, D; D, non-D; and non-D, non-D) is given for each test.

^†^ Analyzed only for allele sizes that were present in ≥ 4% of the population.

*P value for allele 3.7 kb was not significant following correction for 44 tests (p=0.4).
