# Supplementary material for: Mucin Variable Number Tandem Repeat Polymorphisms and Severity of Cystic Fibrosis Lung Disease: Significant Association with MUC5AC
Source: PLoS One. 2011 Oct 6;6(10):e25452. doi: 10.1371/journal.pone.0025452 (PMC3188583; doi:10.1371/journal.pone.0025452)
Supplement: Table S3 — Association of MUC1 , MUC2 and MUC5AC SNPs with CF lung phenotype. (DOC) [file pone.0025452.s007.doc]

**Table S3.** **Association of *MUC1*, *MUC2* and *MUC5AC* SNPs with CF lung phenotype*.**

|  |  | **Number of patients** | | |  |
| --- | --- | --- | --- | --- | --- |
|  |  | **Severe** | **Mild** |  | **P Value**** |
| **Gene** | **SNP** | **n (%)** | **n (%)** | **Total** | **(nominal)** |
| *MUC1* | rs11465212 | 242 (31.5) | 527 (68.5) | 769 | 7.9 x 10^-1^ |
|  | rs1611770 | 242 (31.4) | 529 (68.6) | 771 | 10 x 10^-1^ |
|  | rs11465205 | 242 (31.4) | 528 (68.6) | 770 | 7.6 x 10^-1^ |
| *MUC2* | rs10902076 | 242 (31.4) | 528 (68.6) | 770 | 4.5 x 10^-3^† |
|  | rs2856111 | 242 (31.4) | 529 (68.6) | 771 | 4.0 x 10^-1^ |
|  | rs7952257 | 242 (31.4) | 529 (68.6) | 771 | 9.0 x 10^-1^ |
|  | rs7944723 | 217 (40.3) | 322 (59.7) | 539 | 2.1 x 10^-1^ |
|  | rs10902089 | 242 (31.4) | 529 (68.6) | 771 | 3.2 x 10^-1^ |
|  | rs10794293 | 241 (31.4) | 527 (68.6) | 768 | 2.2 x 10^-1^ |
|  | rs7952385 | 242 (31.5) | 527 (68.5) | 769 | 8.9 x 10^-1^ |
| *MUC5AC* | rs17859811 | 242 (31.4) | 529 (68.6) | 771 | 6.5 x 10^-1^ |
|  | rs2075842 | 241 (31.4) | 527 (68.6) | 768 | 2.8 x 10^-1^ |
|  | rs3087562 | 242 (31.4) | 529 (68.6) | 771 | 2.1 x 10^-1^ |
|  | rs13380 | 242 (31.4) | 529 (68.6) | 771 | 1.8 x 10^-1^ |
|  | rs28472384 | 242 (31.4) | 529 (68.6) | 771 | 2.4 x 10^-1^ |

* SNP genotypes determined by ABI TaqMan probes or Illumina GoldenGate technology

**Logistic Regression, using covariates of gender and principal components (n=10)

† Bonferroni corrected p value for 15 tests, p=0.068
