# Supplementary material for: Mucin Variable Number Tandem Repeat Polymorphisms and Severity of Cystic Fibrosis Lung Disease: Significant Association with MUC5AC
Source: PLoS One. 2011 Oct 6;6(10):e25452. doi: 10.1371/journal.pone.0025452 (PMC3188583; doi:10.1371/journal.pone.0025452)
Supplement: Table S4 — Two-variant haplotype analysis for 6.3 and 6.4 kb MUC5AC VNTR alleles with flanking SNPs. (DOC) [file pone.0025452.s008.doc]

**Table S4. Two-variant haplotype analysis for 6.3 and 6.4 kb *MUC5AC* VNTR alleles with flanking SNPs*.**

| **Variant 1** | **Variant 2** | **P Value**** |
| --- | --- | --- |
| rs10902096 | rs7112954 | 3.9 x 10^-1^ |
| rs10902096 | rs11245979 | 6.6 x 10^-1^ |
| rs10902096 | *MUC5AC*(6.3) | 9.0 x 10^-2^ |
| rs10902096 | *MUC5AC*(6.4) | 6.7 x 10^-4^ |
| rs10902096 | rs35705491 | 7.7 x 10^-1^ |
| rs10902096 | rs28514396 | 8.1 x 10^-1^ |
| rs10902096 | rs28678421 | 8.5 x 10^-1^ |
| rs10902096 | rs7120886 | 7.9 x 10^-1^ |
| rs7112954 | rs11245979 | 7.0 x 10^-1^ |
| rs7112954 | *MUC5AC*(6.3) | 1.0 x 10^-1^ |
| rs7112954 | *MUC5AC*(6.4) | 4.2 x 10^-4^ |
| rs7112954 | rs35705491 | 5.5 x 10^-1^ |
| rs7112954 | rs28514396 | 8.2 x 10^-1^ |
| rs7112954 | rs28678421 | 8.7 x 10^-1^ |
| rs7112954 | rs7120886 | 8.3 x 10^-1^ |
| rs11245979 | *MUC5AC*(6.3) | 1.2 x 10^-1^ |
| rs11245979 | *MUC5AC*(6.4) | 1.1 x 10^-4^ |
| rs11245979 | rs35705491 | 7.8 x 10^-1^ |
| rs11245979 | rs28514396 | 8.3 x 10^-1^ |
| rs11245979 | rs28678421 | 3.8 x 10^-1^ |
| rs11245979 | rs7120886 | 4.9 x 10^-1^ |
| *MUC5AC*(6.3) | *MUC5AC*(6.4) | 1.9 x 10^-4^ |
| *MUC5AC*(6.3) | rs35705491 | 5.0 x 10^-2^ |
| *MUC5AC*(6.3) | rs28514396 | 4.2 x 10^-2^ |
| *MUC5AC*(6.3) | rs28678421 | 1.1 x 10^-1^ |
| *MUC5AC*(6.3) | rs7120886 | 3.2 x 10^-2^ |
| *MUC5AC*(6.4) | rs35705491 | 2.5 x 10^-4^ |
| *MUC5AC*(6.4) | rs28514396 | 5.2 x 10^-4^ |
| *MUC5AC*(6.4) | rs28678421 | 1.0 x 10^-3^ |
| *MUC5AC*(6.4) | rs7120886 | 5.5 x 10^-4^ |
| rs35705491 | rs28514396 | 4.0 x 10^-1^ |
| rs35705491 | rs28678421 | 8.1 x 10^-1^ |
| rs35705491 | rs7120886 | 9.4 x 10^-1^ |
| rs28514396 | rs28678421 | 9.5 x 10^-1^ |
| rs28514396 | rs7120886 | 9.0 x 10^-1^ |
| rs28678421 | rs7120886 | 6.6 x 10^-1^ |

*Analysis was done in R using haplo.score.

**For haplotype analysis, each combination was computed by 100,000 permutations.
